# Supplementary material for: The genome sequence of a hoverfly , Melangyna compositarum (Verrall, 1873) (Diptera: Syrphidae)
Source: Wellcome Open Res. 2026 Mar 18;11:179. [Version 1] doi: 10.12688/wellcomeopenres.26158.1 (PMC13069379; doi:10.12688/wellcomeopenres.26158.1)
Supplement: Peer review history

# Reviewer report: Reviewer response for version 1

**DOI:** 10.21956/wellcomeopenres.28807.r151905

**Published:** 2026-04-10

**Authors:** Hans-Peter Fuehrer

**Affiliations:** 1 University of Veterinary Medicine, Veinna, Austria

The authors analyzed the genome of a hoverfly, Melangyna compositarum (Verrall, 1873) (Diptera, Syrphidae). The biology of the species and the difficulties involved in morphological identification are discussed. The manuscript is of good quality and only minor modifications are recommended.

 The genus is known as “Spot-tails.”

 How was the specimen collected?

Are sufficient details of methods and materials provided to allow replication by others?

Yes

Is the rationale for creating the dataset(s) clearly described?

Yes

Are the datasets clearly presented in a useable and accessible format?

Yes

Are the protocols appropriate and is the work technically sound?

Yes

Reviewer Expertise:

Medical entomology

I confirm that I have read this submission and believe that I have an appropriate level of expertise to confirm that it is of an acceptable scientific standard.

# Reviewer report: Reviewer response for version 1

**DOI:** 10.21956/wellcomeopenres.28807.r152479

**Published:** 2026-04-08

**Authors:** Darren Obbard

**Affiliations:** 1 The University of Edinburgh, Edinburgh, Scotland, UK

This data note reports the sequencing and assembly of the genome of Melangyna compositarum as part of the “Darwin Tree of Life” programme. In common with other data notes from this research effort, the reporting is standardised and quite brief. As such, I have very few comments to make.

 The approach is state-of-the-art, the raw data appear to be of a suitably high quality, and the assembly methods are appropriate. The public availability of raw data and genome assembly are appropriate. The resulting genome is likely to be of very high quality, and I have no doubt that it will be of great value to any researchers working on syrphids and other diptera, or on the comparative or evolutionary genomics of insects more generally.

 My minor suggestions are:

 (1) There is an extreme typo / copy-paste error near the start, when Melangyna labiatarum is written as Melissodes labiatarum (that would be a bee, I think?)

 (2) In addition to the sequenced specimen itself, it would be nice to have an ‘in life’ photograph and/or perhaps both sexes. Perhaps even a good photo of the (subtle!) differences between M. compositarum and M. labiatarum.   

 (3) I think the reporting of the taxonomic status is a little too brief. It is interesting to know that “Some debate exists around whether they truly represent separate species (Ball & Morris, 2015; Stubbs & Falk, 2002).” But those two references are of little value to most readers, who (not being based in the UK) are unlikely to have access to UK field guides. What is the gist of those argument? What genetic data have been used to support either side, or is it purely morphological? Does the debate extend to taxonomists outside of the UK? I think this warrants at least another 3 sentences, especially given the potential value of a genome in resolving the argument

 (4) I am surprised that no mention is made of the potential value of this genome in resolving the taxonomic debate. Obviously, a genome might tell us nothing – but equally, it could resolve the question instantly! It is a shame that DToL do not have labiatarum …

 (5) has anyone proposed common names for this species? If so, what?

 (6) Style: Please avoid starting a sentence with “M.”

Are sufficient details of methods and materials provided to allow replication by others?

Yes

Is the rationale for creating the dataset(s) clearly described?

Yes

Are the datasets clearly presented in a useable and accessible format?

Yes

Are the protocols appropriate and is the work technically sound?

Yes

Reviewer Expertise:

Evolutionary genetics and genomics of Diptera and their pathogens

I confirm that I have read this submission and believe that I have an appropriate level of expertise to confirm that it is of an acceptable scientific standard.

# Reviewer report: Reviewer response for version 1

**DOI:** 10.21956/wellcomeopenres.28807.r151910

**Published:** 2026-04-08

**Authors:** Jaakko Pohjoismäki

**Affiliations:** 1 University of Eastern Finland, Joensuu, Finland

The presented work by Liam M. Crowley, Katie J. Woodcok and the DToL consortium presents the assembly and basic characterization of the hoverfly *Melangyna compositarum* genome, following established high-throughput sequencing and assembly pipelines of the Darwin Tree of Life Project. Overall, the manuscript is clear, technically sound, and meets the expected standards.

 The genome assembly is of high quality and continuity. The combination of PacBio long-read sequencing and Hi-C scaffolding has produced a highly contiguous assembly with a chromosome-scale scaffold N50 (253 Mb) and a high proportion (91.7%) of sequence assigned to chromosomes. Consensus quality values (QV ~57) and BUSCO completeness (98.6%) indicate a near-complete and accurate representation of the gene space. The sequencing depth (40x) is more than sufficient given the genome size and complexity. Also, the haplotypes have been resolved.

 I have only a couple of minor points:

 The duplicated BUSCO proportion (8.0%) is a bit above the recommended benchmark (<5%), which may reflect residual haplotypic duplication or assembly artifacts; a brief comment on this would improve transparency. Similarly, the relatively low k-mer completeness for individual haplotypes (77% and 70%) compared to the combined assembly suggests some fragmentation. The absence of an identifiable Y chromosome is noted. Sex chromosomes appear to be tricky but have been identified for other brachyceran flies by read coverage, heterochromatic region size and BUSCO (diptera_odb12) gene count. These points could be briefly discussed, but this is not absolutely essential as future comparative analyses might want to address such issues more universally.

 However, there is a bit awkward error in the Background section that should be corrected: *Melissodes* is a Nearctic bee genus. *Melangyna labiatarum* does exists and fits the context here, so easy to fix.

 Otherwise, this is a solid and valuable genome resource that will be useful for future studies on hoverfly genetics and comparative genomics.

Are sufficient details of methods and materials provided to allow replication by others?

Yes

Is the rationale for creating the dataset(s) clearly described?

Yes

Are the datasets clearly presented in a useable and accessible format?

Yes

Are the protocols appropriate and is the work technically sound?

Yes

Reviewer Expertise:

cell & molecular biology, biodiversity genomics, taxonomy

I confirm that I have read this submission and believe that I have an appropriate level of expertise to confirm that it is of an acceptable scientific standard, however I have significant reservations, as outlined above.
